# School situation for specially abled children – How can we as qualitative researchers contribute?

**DOI:** 10.3402/qhw.v9.24396

**Published:** 2014-04-16

**Authors:** Ulrika Hallberg

**Affiliations:** Nordic School of Public Health (NHV), Gothenburg, Sweden

School is and should be an important part in the lives of all children and youth. Much time is spent in school, and it serves not only as an educational arena but also as a social arena that fosters us as human beings. Lifelong friendships are ultimately developed in school, which serve as a protection when life gets rough. Our achievements in school predict our later possibilities to attend higher education and in turn possibilities to find a job, which has an influence on our social and financial standard, as also our health and wellbeing.

Many countries in the Western world are actively working toward the inclusion of specially abled children in schools primarily planned and built for children without disabilities. This is a political standpoint based on every individual's equal right to participate in the society on equal terms. There is an ongoing debate in the research society as to whether this is in the best interest of the child with a disability. We know today that there are both benefits and risks in including these children in “regular” schools. We also know that if the child's individual needs are not fulfilled, then inclusion in “regular” schools is not fruitful for any individual involved, especially not for the child with a disability.

The intention of including specially abled children in “regular” schools is primarily to give these children a chance to socialize and build long-lasting relationships with children without disabilities. Having friends is crucial for development in all children. It is within these relationships that socialization takes place, and important values and approaches such as respect toward others are shaped and tried out. Long-lasting friendships also serve as a protection when children are going through rough times in life. Another benefit in the inclusion of specially abled children in “regular” schools is to acknowledge them in society, which in the long term leads to increased acceptance for this group.

Sadly, many specially abled children who are included in “regular” schools are excluded from the possibilities of creating friendships with other children without disabilities. Often, their schoolmates have a neutral or indifferent attitude toward them, sometimes even a negative attitude. In worst cases the specially abled children are teased or even bullied. Bullying in childhood can lead to decreased self-esteem, social isolation, anxiety, and suicidal thoughts both in the short and long term. This means that creating friendships in children with and without disabilities is not done automatically; instead it requires an active engagement from adults around the children. Specially abled children often absent from school because of their disabilities. It is difficult for the teachers, who often lack special training, to help these children to make up for their absence which means such children often underperform compared with their normal schoolmates. This also affects their self-esteem.

On the contrary, specially abled children who attend schools built specifically to cater to their special needs often have better self-esteem. They mirror themselves with other children who also have special needs which mean that they, to some extent, are happily unaware of their individual shortcomings, which in turn serve as a protection from decreased self-esteem. Their teachers often have special training to understand and appreciate their needs, thereby willing to impart the lessons in accordance with the individual child's level of understanding and needs.

We know that specially abled children themselves regard negative attitudes from schoolmates as the biggest obstacle for inclusion in “regular” schools. But we need more knowledge to help these children to increase their self-esteem and their achievements in school. Here qualitative researchers have an important role to play. High-quality qualitative studies are an excellent instrument in expanding the knowledge about these children's school situations and thereby improving their lives both in the short and long term. To let the children themselves speak and tell their stories can give us valuable insight into their situation and can guide us in our attempts to improve the health and wellbeing of all specially abled children.

*Ulrika Hallberg* Associate Professor Nordic School of Public Health (NHV) Gothenburg, Sweden

